# Guselkumab: widened action in psoriatic disease

**DOI:** 10.6061/clinics/2021/e2629

**Published:** 2021-04-26

**Authors:** Marcelo Arnone, André Vicente Esteves de Carvalho, Lincoln Zambaldi Fabricio, Ricardo Romiti

**Affiliations:** I Departamento de Dermatologia, Hospital das Clinicas HCFMUSP, Faculdade de Medicina, Universidade de Sao Paulo, Sao Paulo, SP, BR; II Hospital Moinhos de Vento, Porto Alegre, RS, BR; III Servico de Dermatologia, Faculdade Evangelica Mackenzie do Parana, Curitiba, PR, BR

Immunobiological therapy has been one of the greatest accomplishments in the field of medicine in recent years, leading to the improvement of the quality of life and clinical improvement in millions of patients. Physiopathogenesis has been extensively studied and dermatologists have played an important role in investigating this process. Therapeutic innovations arrived for psoriasis patients in a secondary way, where comorbidities had the treatment rationale for themselves. Fortunately, the priorities of immunobiological research changed this perspective, leading to the development of several new drugs for dermatological diseases as the first-line treatment.

Blockade of interleukin (IL)-23 pro-inflammatory cytokine by anti-IL-23 specific sub-unit p19 monoclonal antibody, guselkumab, resulted in 4 years of safe and excellent sustained therapeutic effects in patients with moderate-to-severe psoriasis (1). The Lancet, March 2020, published two new studies (see below) describing the advancement of dermatology with the first anti-IL23 immunobiological drug approved for use in rheumatology (2,3).

In March 2018, guselkumab was the first IL-23p19 antagonist that was approved for plaque psoriasis treatment in Brazil (4). In psoriasis, pivotal phase III studies VOYAGE 1 and 2 (multicenter, randomized, double-blind, with adalimumab as an active comparator, and placebo) showed a significantly higher response rate in patients receiving guselkumab, with PASI75, PASI90, and PASI100 responses within 48 weeks, than in those receiving adalimumab and placebo (2). In the NAVIGATE study assessing the efficacy and safety of guselkumab in patients with moderate-to-severe plaque psoriasis who did not respond to ustekinumab treatment, the mean decrease in PASI from week 16 was significantly higher in the guselkumab group at week 52, compared to the group that remained in the ustekinumab group (6.7 *vs.* 2.5%); patients treated with ustekinumab who did not reach an IGA of 0/1 at week 16 were significantly benefitted after switching to guselkumab (5). In the comparative study with secukinumab named ECLIPSE, the rate of patients with PASI90 response at week 48 was higher in the guselkumab group (84.5%) than in the secukinumab group (70%), while PASI100 response was 58.2% and 48.4% respectively (2). The analysis of response to guselkumab in patients with severe psoriasis forms, such as generalized pustular psoriasis, as well as in special areas such as the scalp, palms, soles, and nails, also showed favorable outcomes (5).

The efficacy and safety of guselkumab in patients with active psoriatic arthritis were reported in two randomized, phase 3, multicenter, double-blind, placebo-controlled clinical trials (DISCOVER-1 and DISCOVER-2) that had as a primary endpoint of ACR20 response at week 24 (2,3). The studies assessed a total of 1122 patients assigned to three groups: 100 mg guselkumab at weeks 0 and 4 and maintenance every 8 weeks, 100 mg guselkumab every 4 weeks, and placebo. In DISCOVER 1, patients previously on anti-TNF-α were enrolled, and in DISCOVER 2, only biologically naïve patients were enrolled. In both studies, the rate of patients who reached the ACR20 response was significantly higher in groups treated with guselkumab than in those treated with placebo. Responses in DISCOVER-1 were 59% in the guselkumab group every 4 weeks, 52% in the guselkumab group every 8 weeks, and 22% in the placebo group. Responses in DISCOVER-2 were 64% in the guselkumab group every 4 weeks, 63% in the guselkumab group every 8 weeks, and 33% in the placebo group (Figure 1). The safety profile was deemed satisfactory, without any opportunistic infection or tuberculosis.

Anti-IL23 immunobiologicals raise important questions regarding the specificity of the choice of treatment for psoriasis. The high efficacy in psoriasis treatment, with response levels higher than anti-TNFs, the comparable response to the same drugs in the treatment of the articular manifestations and the class safety, with no indication of influence on the incidence of tuberculosis (6), leads to the conclusion that anti-IL23 antibody may be the medication of choice for the treatment of several psoriasis manifestations. The absence of class-specific adverse events so far may be an advantage for patients with a contraindication to the use of anti-IL17 immunobiologicals. Considering tuberculosis is endemic to Brazil, anti-IL23 could be a safer and more efficient alternative for the first-choice treatment of moderate-to-severe psoriasis, associated or not with psoriatic arthritis with appendicular manifestation, than anti-TNFs (6). In conclusion, new immunobiologicals including anti-IL23 are relevant to clinical dermatology, provide a new physiopathology insight into inflammatory and neoplastic diseases, and represent a more efficient and safer alternative to anti-TNFs for the treatment of skin and joint manifestations of psoriatic disease.

## Conflicts of Interest

MA is/has served as a scientific consultant, speaker, or clinical study investigator for Abbvie, Glenmark, Janssen, Lilly, Leo-Pharma Novartis, Pfizer, and UCB. AC is/has served as a scientific consultant, speaker, or clinical study investigator for Abbvie, Boehringer-Ingelheim, Janssen, Lilly, Leo-Pharma, Novartis, and UCB. RR is/has served as a scientific consultant, speaker, or clinical study investigator for AbbVie, Boehringer, Galderma, Janssen-Cilag, Lilly, Leo-Pharma, Novartis, Pfizer, TEVA, and UCB. LF is/has served as a scientific consultant, speaker, or clinical study investigator for Abbvie, Aché, Bayer, Bioderma, Galderma, Hypermarcas, Isdin, Janssen, La Roche-Posay, Leo-Pharma, Novartis, Pfizer, Sanofi, Stieffel/Gsk, and UCB.

## AUTHOR CONTRIBUTIONS

Arnone M, Carvalho AVE, Fabricio LZ and Romiti R contributed to the manuscript drafting and critically review for important intellectual content, and were also responsible for the approval of the final version to be submitted.

## Figures and Tables

**Figure 1:**
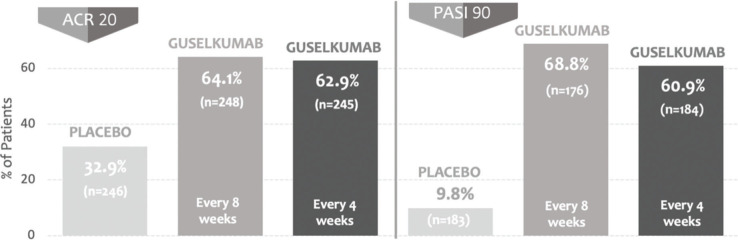
Main endpoints of interest (DISCOVER-2) (3).

## References

[1] Griffiths CEM, Papp K, Song M, Randazzo B, Li S, Shen YK (2018). Maintenance of Response With Guselkumab for up to 3 Years’ Treatment in the Phase 3 VOYAGE 1 Trial of Patients With Plaque Psoriasis. Poster presented at: Falls Clinical Dermatology Conference. SKIN The Journal of Cutaneous Medicine.

[2] Deodhar A, Helliwell PS, Boehncke WH, Kollmeier AP, Hsia EC, Subramanian RA (2020). Guselkumab in patients with active psoriatic arthritis who were biologic-naive or had previously received TNFα inhibitor treatment (DISCOVER-1): a double-blind, randomised, placebo-controlled phase 3 trial. Lancet.

[3] Mease PJ, Rahman P, Gottlieb AB, Kollmeier AP, Hsia EC, Xu XL (2020). Guselkumab in biologic-naive patients with active psoriatic arthritis (DISCOVER-2): a double-blind, randomised, placebo-controlled phase 3 trial. Lancet.

[4] Tremfya [package insert] (2019). Horsham (PA): Janssen Biotech, Inc.

[5] Puig L (2019). Guselkumab for the treatment of adults with moderate to severe plaque psoriasis. Expert Rev Clin Immunol.

[6] Nogueira M, Warren RB, Torres T (2021). Risk of tuberculosis reactivation with interleukin (IL)-17 and IL-23 inhibitors in psoriasis - time for a paradigm change. J Eur Acad Dermatol Venereol.

